# Unilateral absence of the thyrocervical trunk with multiple subclavian arterial variations and a suprascapular artery traversing the brachial plexus: a cadaveric case report

**DOI:** 10.1007/s00276-026-03992-x

**Published:** 2026-09-10

**Authors:** Katherine Asbery, Lauren Ellingham, Arunabh Bhattacharya

**Affiliations:** 1 University of the Incarnate Word School of Osteopathic Medicine, 7615 Kennedy Hill Drive, San Antonio, TX USA; 2 Department of Applied Biomedical Sciences, University of the Incarnate Word School of Osteopathic Medicine, 7615 Kennedy Hill Drive, San Antonio, TX 78235 USA; 3 Division of Anatomy and Molecular Medicine, Alabama College of Osteopathic Medicine, 445 Health Sciences Blvd, Dothan, AL 36303 USA

**Keywords:** Thyrocervical trunk, Suprascapular artery, Transverse cervical artery, Inferior thyroid artery, Brachial plexus

## Abstract

**Purpose:**

Understanding anatomical variations in the branching pattern of the subclavian artery (SA) is critical to reducing complications during surgical and interventional procedures involving the neck and shoulder regions. This cadaveric study reports a rare combination of vascular anomalies involving the branches of the SA.

**Methods:**

Bilateral dissections of the neck, shoulder, and upper back were performed on a 71-year-old Caucasian male donor to document the origin, course, and distribution of the branches of the SA.

**Results:**

Multiple unilateral variations were observed on the right side of the donor. The first variation noted was the absence of the thyrocervical trunk, with its typical branches showing variable origins. Inferior thyroid artery originated with the vertebral artery from the thyrovertebral trunk, representing the second variation. Transverse cervical artery was completely absent, representing the third variation. The fourth variation was the dorsal scapular artery serving as the primary blood supply to the trapezius muscle. The suprascapular artery (SSA) originated from the axillary artery and coursed posteriorly between the anterior divisions of the upper and middle trunks, immediately superior to their union to form the lateral cord, representing the fifth variation. Lastly, the SSA entered the supraspinous fossa beneath the superior transverse scapular ligament, accompanied by the suprascapular nerve, representing the sixth variation.

**Conclusion:**

This case demonstrates a unique combination of six arterial variations that, to our knowledge, have not been previously reported together. Awareness of such variants is important for surgical planning and may help reduce intraoperative complications in head, neck, and shoulder procedures.

## Introduction

The thyrocervical trunk (TCT) and its branches provide blood supply to structures in the neck, shoulder, and upper back. Classically, the TCT arises from the first part of the subclavian artery (SA), medial to the anterior scalene muscle (ASm) and gives rise to four branches: the inferior thyroid artery (ITA), the transverse cervical artery (TCA), the suprascapular artery (SSA) and the ascending cervical artery (ACA) [8, 18]. The TCA is the primary arterial supply to the trapezius muscle [11]. The ITA provides arterial supply to the structures within the endocrine, respiratory and alimentary layers of the neck [6]. The SSA is the primary blood supply to two of the four rotator cuff muscles, supraspinatus and infraspinatus [6]. The dorsal scapular artery (DSA) typically arises from the third part of the SA lateral to the ASm and serves as the major arterial supply to the rhomboid major, rhomboid minor and levator scapulae muscles, with a minor contribution to the trapezius muscle [11, 18].

The branches of the SA exhibit considerable variation in both their origin and course [13, 25, 33]. Recognition of these variations is important for surgical planning and for reducing the risk of vascular and neural injury during procedures involving the neck and shoulder regions [4, 16, 29]. Although variations in the origin and course of the SSA have been reported relatively frequently [29], its origin from the axillary artery (AA), its course between the anterior divisions of the upper and middle trunks of the brachial plexus (BP), and its passage beneath the superior transverse scapular ligament (STSL) together with the suprascapular nerve (SSN) are uncommon [22, 28]. Similarly, absence of the TCT and TCA, the DSA serving as the primary blood supply to the trapezius muscle, and the origin of the ITA from the thyrovertebral trunk (TVT) have each been rarely reported in the literature [14, 16, 28, 33]. To the best of our knowledge, previous reports have not described the coexistence of these arterial variations in a single case. This case expands the existing literature on SA branching patterns and highlights the importance of recognizing concurrent vascular variations during surgical and interventional procedures involving the neck and shoulder.

## Case report

Bilateral dissections were performed on the neck, shoulder and back of a 71-year-old Caucasian male donor. The donor was obtained through the Willed Body Program at the University of Texas Health at San Antonio for the purposes of medical education and research. The skin, subcutaneous tissue, and superficial fascia of the anterior and lateral neck regions were removed. Following removal of the investing layer of the deep cervical fascia, the sternocleidomastoid muscle was reflected to expose the underlying muscles and neurovascular structures.

**Fig. 1:**
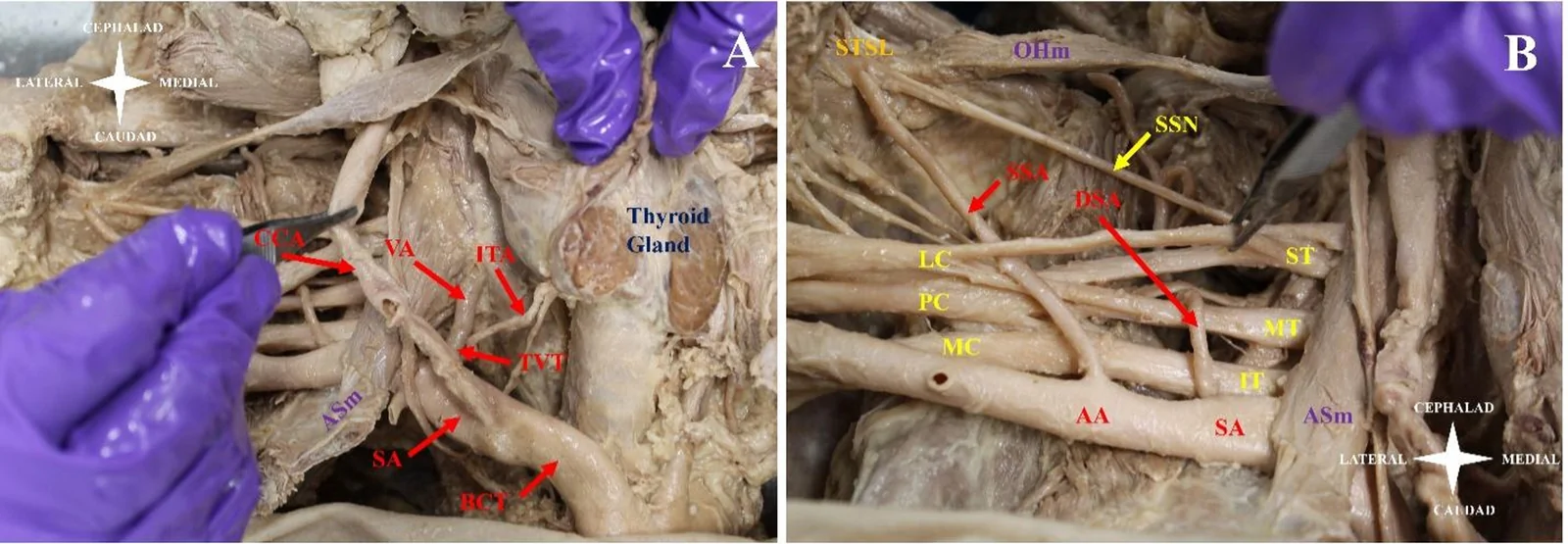
Anterior view of the right side of the neck. **A** Vertebral and inferior thyroid arteries arose from the thyrovertebral trunk from the subclavian artery. The common carotid artery was retracted to the right for clarity. **B** Suprascapular artery arose from the axillary artery and coursed posteriorly between the anterior divisions of the upper and middle trunks of the brachial plexus. AA: axillary artery, ASm: anterior scalene muscle, BCT: brachiocephalic trunk, CCA: common carotid artery, DSA: dorsal scapular artery, IT: inferior trunk, ITA: inferior thyroid artery, LC: lateral cord, MC: medial cord, MT: middle trunk, OHm: omohyoid muscle, PC: posterior cord, SA: subclavian artery, SSA: suprascapular artery, SSN: suprascapular nerve, STSL: superior transverse scapular ligament, ST: superior trunk, TVT: thyrovertebral trunk, VA: vertebral artery

**Fig. 2:**
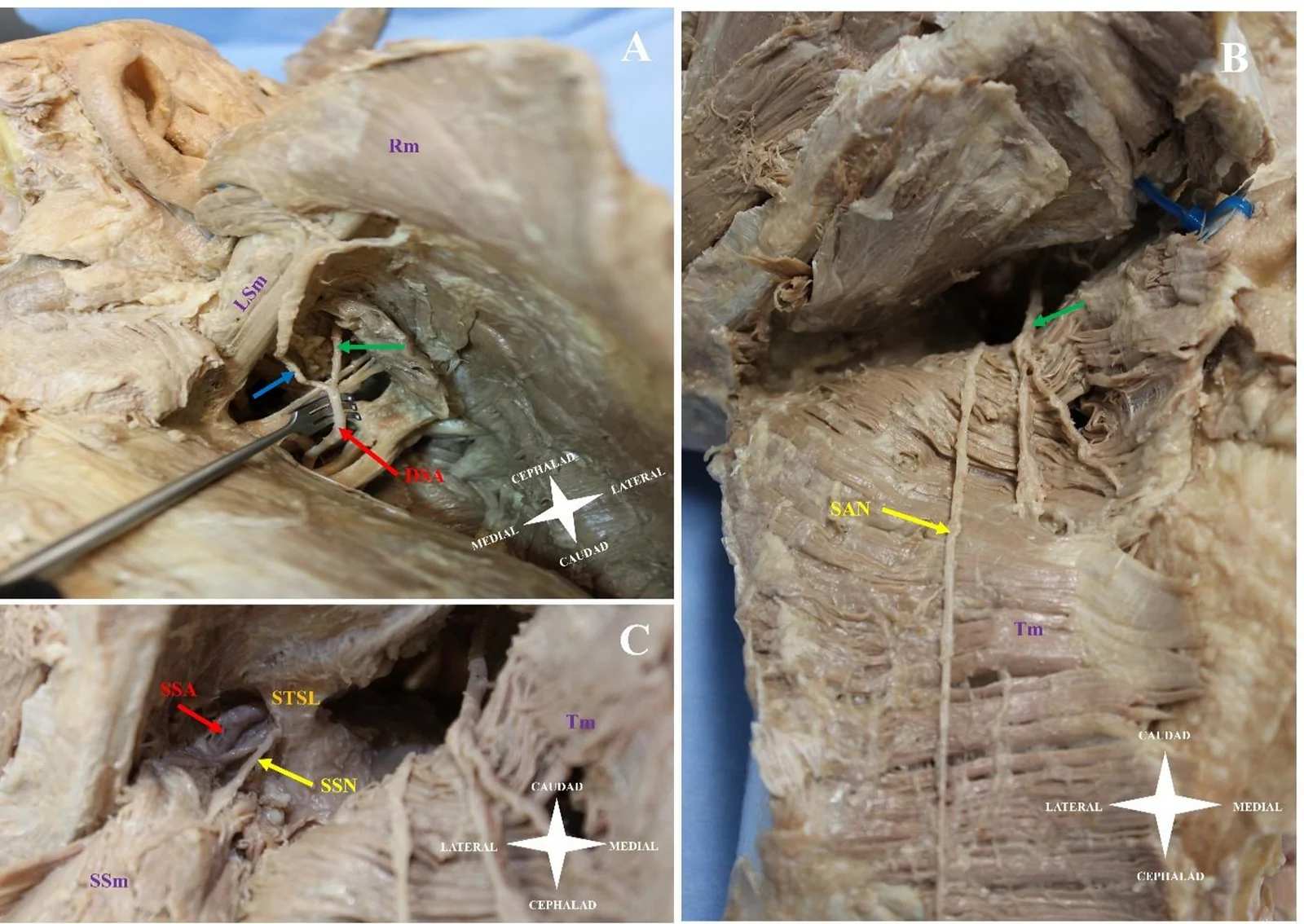
Posterior view of the muscles and vasculature of the scapular region and upper back. **A** Posterior view of scapular region shows branching of the dorsal scapular artery. Blue and green arrows indicate dorsal scapular artery branches to levator scapulae and rhomboid muscles, and trapezius muscles, respectively. **B** Branch of dorsal scapular artery supplying the trapezius muscle (reflected). **C** Superior view of the supraspinous fossa showing the passage of suprascapular artery and nerve, inferior to the superior transverse scapular ligament. DSA: dorsal scapular artery, LSm: levator scapulae muscle, Rm: rhomboid muscles, SAN: spinal accessory nerve, SSA: suprascapular artery, STSL: superior transverse scapular ligament, SSm: supraspinatus muscle, SSN: suprascapular nerve, Tm: trapezius muscle

The first variation identified was the absence of the right TCT, with its typical branches showing variable origins (Fig. 1A, B). A TVT was present, giving rise to the ITA and the vertebral artery, representing the second variation (Fig. 1A). The third variation noted was the complete absence of the right TCA. Because TCA was absent, the back was dissected and the right trapezius muscle reflected to determine its arterial supply. The right DSA, which arose normally from the third part of the SA, served as the primary arterial supply to the trapezius muscle, representing the fourth variation (Figs. 1A and 2A and B). The SSA originated from the AA and coursed posteriorly, initially passing superficial to the anterior division of the inferior trunk of the BP and thereafter between the anterior divisions of the upper and middle trunks, immediately superior to their union to form the lateral cord, representing the fifth variation (Fig. 1A). The right SSA entered the supraspinous fossa beneath the STSL, accompanied by the SSN, representing the sixth variation (Fig. 2C). TCT and its branching were typical on the left side of the donor.

## Discussion

The SAs begin to develop during the seventh week of embryogenesis. The right SA is derived from the right fourth aortic arch, the right dorsal aorta, and the right seventh intersegmental artery, whereas the left SA develops primarily from the left seventh intersegmental artery [26]. During this period, longitudinal anastomoses between the cervical intersegmental arteries contribute to the formation of the branches of the SA, including the ACA, TCA, SSA and ITA, which typically organize into the TCT [12]. Variations in the branching pattern of these vessels are thought to result from persistence of embryonic vascular channels that normally regress, regression of vessels that normally persist or selective enlargement of alternative channels within the primitive cervical vascular plexus [3]. In the present case, these developmental mechanisms may explain the absence of the TCT and TCA, persistence of the TVT, anomalous origin of SSA from the AA, and the DSA serving as the dominant arterial supply to the trapezius muscle. Although the precise embryological events responsible for this constellation of findings cannot be determined, persistence and regression of alternative embryonic vascular pathways provide a plausible developmental basis for the concurrent arterial variations observed in the present case.

Variations in the branching pattern of TCT are common, whereas complete absence of the trunk has been rarely reported. Recent imaging and cadaveric studies have demonstrated considerable variability in TCT branching, with the most common patterns involving differences in the origin and arrangement of its branches rather than complete absence of the trunk [4, 21]. In a systematic review of SA abnormalities in 1,118 cadaveric specimens, Bruna-Mejias et al. did not identify any cases of an absent TCT [4]. Similarly, Ostrowski et al. did not find any example of absent TCT in a computed tomography angiography (CTA) study of 41 patients [21]. In contrast, Lopez-Muniz et al., Kumar et al. and Yucel et al. each reported cases of an absent TCT [15, 19, 33]. Consistent with these reports, our donor demonstrated unilateral absence of the TCT on the right side together with additional arterial variations.

Complete absence or an incomplete TCT is often accompanied by variability in the origin of its branches, including the ITA. Buena et al. reported direct origin of ITA from the SA in 31.48% of 108 arteries evaluated by CTA [5]. Lippert and Pabst reported origin of the ITA from the SA medial to ASm in 8% of the cases, whereas origin from the SA lateral to the ASm occurred in only 0.1% of the cases [17]. Kumar et al. described bilateral origin of the ITA from SA in a 70-year-old donor with bilateral absence of TCT [15]. We recently reported unilateral origin of the ITA from the SA in an 87-year-old Caucasian male donor, accompanied by unilateral absence of both the TCA and SSA [28].

Origin of the ITA from the TVT has been rarely reported [10, 14]. Hölbling-Patscheider et al. described a right-sided TVT in a 91-year-old female donor that was associated with absence of the TCA and origin of the SSA from a cervicoscapular trunk [10]. Similarly, Kotil et al. reported a right-sided TVT in an 87-year-old Caucasian male donor [14]. In the same study, TCA, ACA and internal thoracic artery arose from a common trunk on the right side of the body [14]. In the present case, the right ITA originated from the TVT and was associated with the absence of the TCA and the origin of the SSA from the AA, representing a distinct combination of arterial variations.

The TCA is the primary arterial supply to the trapezius muscle with minor branches supplying the levator scapulae and rhomboid muscles [6]. Although variations in the origin of the TCA have been well documented, complete absence of the artery is less common. The TCA has been reported to arise from the second or third part of the SA in 13.2% of the specimens [26], and individual case reports have described additional variant origins, including a common trunk with the ACA and internal thoracic artery [14]. Takafuji et al. reported complete absence of the TCA in 15.3% of the necks examined [30], a finding that has also been documented in individual case reports [15, 28]. In the present case, the right TCA was completely absent, and the DSA served as the dominant arterial supply to the trapezius muscle. To the best of our knowledge, this replacement pattern has been described only once previously in a study from our group [28].

Variations in the origin and course of the SSA have been well documented, with the artery arising most commonly from the TCT and less frequently from the SA, the AA or the subscapular artery [1, 7, 16, 20, 29]. Amongst these variants, origin of the SSA from the AA followed by its passage beneath the STSL together with the SSN has been infrequently reported. This arrangement decreases the area of the suprascapular opening and has been proposed as a potential predisposing factor for SSN entrapment [23]. Naidoo et al. and Polguj et al. reported this configuration in 20 (20 of 100 specimens) and 12.3% (13 of 106 specimens examined), respectively [20, 23]. In contrast, Singh (2018) and Tubbs et al. reported this configuration to be lower, with incidence of 3.6 and 2.5%, respectively [29, 32].

Although variations in the origin of the SSA have been well documented, comparatively few studies have examined its relationship with the BP. In a recent cadaveric study, Lai et al. demonstrated that the course of SSA is closely associated with its arterial origin. SSAs arising from the TCT or the TCA consistently coursed either superficial or deep to the BP, whereas the majority of the SSAs originating from the SA or AA traversed the BP (96%) [16]. Amongst the arteries that passed through the plexus, 50% coursed between the divisions distal to the upper and middle trunks [16]. Dargaud et al. similarly reported SSA passing between the upper and middle trunks of the plexus, although its arterial origin was not documented in the study [7]. In the present case, the SSA originated from the first part of AA and coursed between the anterior divisions of the upper and middle trunks, immediately superior to their union to form the lateral cord before continuing to the suprascapular notch. This observation provides a more precise characterization of the SSA-BP relationship than previous reports and further supports the association between anomalous arterial origin and an altered course through the plexus.

Variations in the branching pattern of the SA may alter expected surgical anatomy and should be recognized during operative planning and interpretation of vascular imaging [13, 25]. Failure to recognize these vascular variations may increase the risk of inadvertent vascular or neural injury during surgical and interventional procedures involving the neck and shoulder [24]. In the present case, the absence of TCA may have implications for cervical dissections and head and neck reconstruction because the TCA is a consistent anatomical landmark and an important recipient artery for free flap reconstruction [27, 31]. Tessler et al. demonstrated that TCA is consistently present in both cadaveric and clinical dissections and suggested that it remains an underutilized recipient vessel for head and neck reconstruction [31]. Consequently, absence of the TCA may necessitate selection of an alternative recipient vessel during vascularized flap planning. Likewise, anomalous origin of the SSA from the AA and its course through the BP may alter the anatomy encountered during supraclavicular approaches, BP surgery, clavicular and shoulder procedures, and angiographic or other endovascular interventions [16, 24, 29]. Because an artery traversing the BP may alter the expected neurovascular relationships and increase the complexity of operative dissection, recognition of this variation is important during surgical exposure and image-guided procedures [16, 29]. Passage of the SSA with the SSN beneath the STSL has been associated with a reduced suprascapular opening and may predispose to SSN entrapment, a recognized cause of shoulder pain, rotator cuff muscle weakness, and muscle atrophy [2, 9, 23]. Although a causal relationship cannot be established from the present cadaveric observation, awareness of these concurrent arterial variations may facilitate surgical planning, improve interpretation of vascular imaging, and aid clinicians in evaluating patients with unexplained shoulder pain or suspected SSN entrapment.

## Conclusion

This case study documents a unique combination of six arterial variations that, to the best of our knowledge, has not been previously reported. Recognition of these concurrent vascular variations may be important for preoperative planning, and surgical and interventional procedures involving the neck and shoulder. Future imaging-based studies are warranted to determine the prevalence and clinical relevance of this combination of arterial variations.

## Data Availability

No datasets were generated or analysed during the current study.
